# Supplementary material for: Outward open conformation of a Major Facilitator Superfamily multidrug/H+ antiporter provides insights into switching mechanism
Source: Nat Commun. 2018 Oct 1;9:4005. doi: 10.1038/s41467-018-06306-x (PMC6167325; doi:10.1038/s41467-018-06306-x)
Supplement: Supplementary file 1 — Supplementary Information [file 41467_2018_6306_MOESM1_ESM.pdf]

Supplementary Information

Outward open conformation of a Major Facilitator Superfamily multidrug/H^+ antiporter provides insights into switching mechanism

Kumar Nagarathinam *et al.*

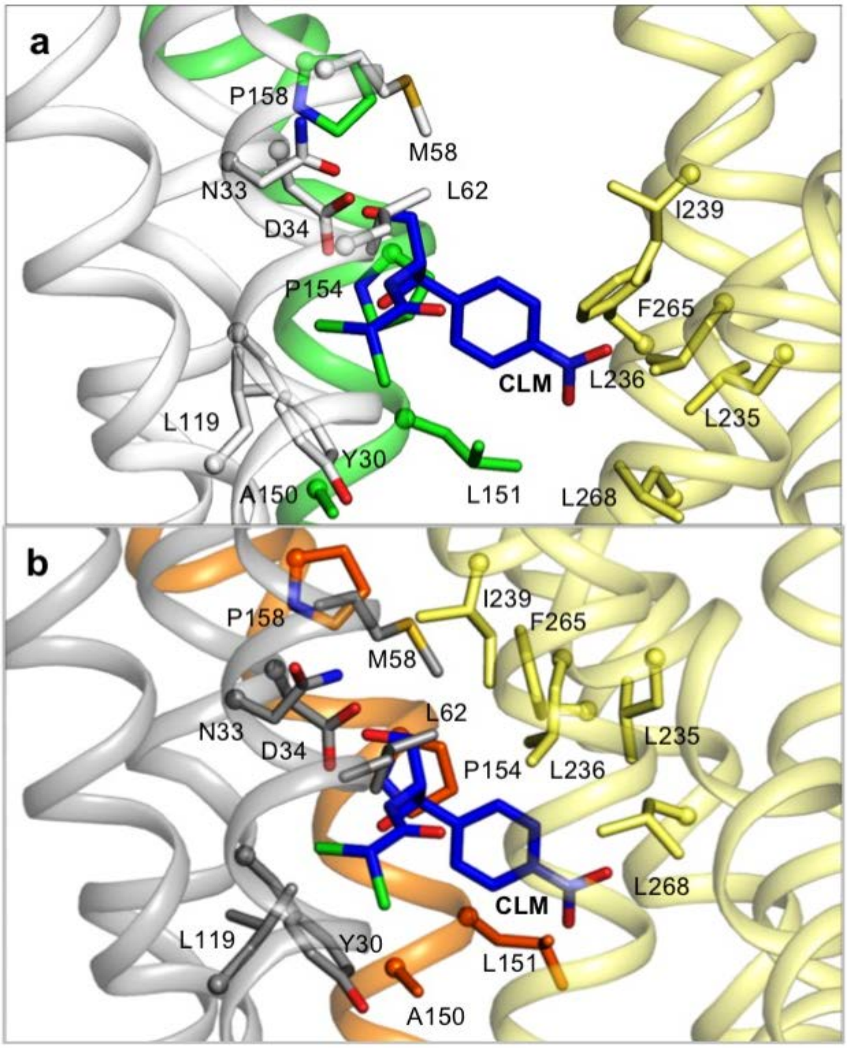

Supplementary Figure 1: The drug binding site is open to the periplasmic space in the outward open conformation (**a;** orientation as in **Figure 1**). The chloramphenicol binding site (obtained by superposition of the N-terminal domain of PDB coordinates 4ZOW[1] in the inward open conformation (**b**) on the present structure) is not only accessible to the periplasm, but is disrupted in the O_o state. Due to a distortion in TM5, N-terminal domain residues $A150^{TM5}$ and $L151^{TM5}$ disengage from the ligand, and rotation of the C-terminal domain results in displacement of residues from TM7 and TM8 by up to 8 Å (Cα – Cα distance).

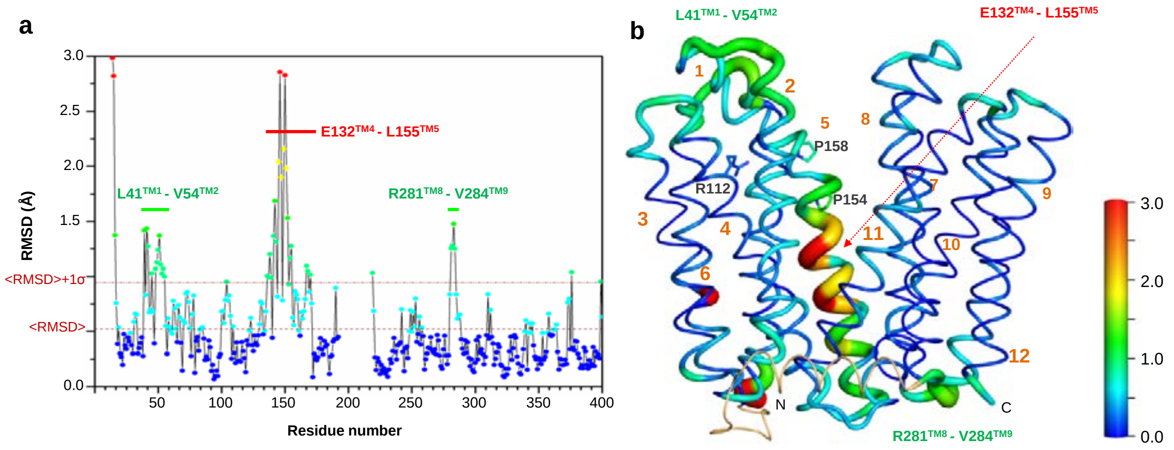

Supplementary Figure 2: Superposition of the individual domains of MdfA in the I_f and O_o conformations reveals significant deviations in the N-terminal domain. (a) Cα distance plots between O_o and I_f states as a function of residue number after separate superposition of the N- and C-terminal domains. Lines marking the mean <RMSD> (0.5211 Å) and <RMSD> + 1σ (σ = 0.4242 Å = standard deviation of RMSD values from the mean) values are shown. Three regions of the sequence show significant deviations between the O_o and I_f structures: Glu132^{TM4}-Leu155^{TM5}, Leu41^{TM1}-Val54^{TM2} and Arg281^{TM8}-Val284^{TM9}. The latter region, which represents the contact site for the TM5 N-terminal residues in the O_o state (see Figure 2), shows structural variation in the I_f conformation in the presence of different ligands[1,2], so that we assume that this exhibits inherent plasticity and does not play a major role in transitioning between the two states. (b) Projection of the Cα distance values per residue on the O_o structure described here using the PyMOL programme. Regions with large and small distances are depicted using thick and thin radii respectively, colored as a spectrum from blue (0.1 Å) to red (3.0 Å).

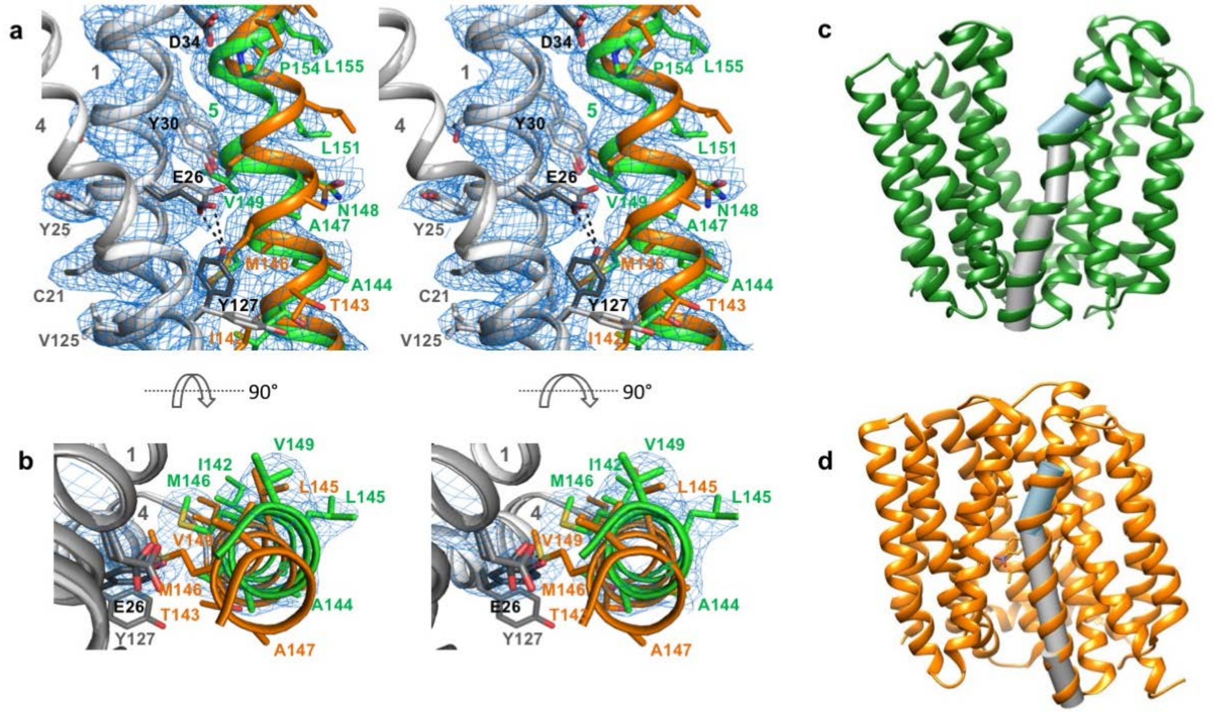

Supplementary Figure 3: Electron densities ($2F_o$-F_c contoured at 1σ, in stereo) for MdfA in the O_o conformation, superimposed with coordinates of the final (O_o, green) and initial (I_f, orange) models. (a) Electron density for TM5 in the O_o conformation, oriented as in **Figure 1**. Overlay of the N-terminal domain from the I_f conformation (light grey, green) fails to satisfy electron density for TM5. **(b)** As in **(a)**, rotated 90° about a horizontal axis. See also accompanying **Supplementary Movie 1**. **(c, d)** Depiction of TM5 helix direction in the O_o (orange) and I_f (green) crystal structures calculated using the program Kink Finder[3]; the two-proline-containing antiporter motif C "153AlaProXaaXaaGlyPro158" of TM5 is shown as a blue cylinder.

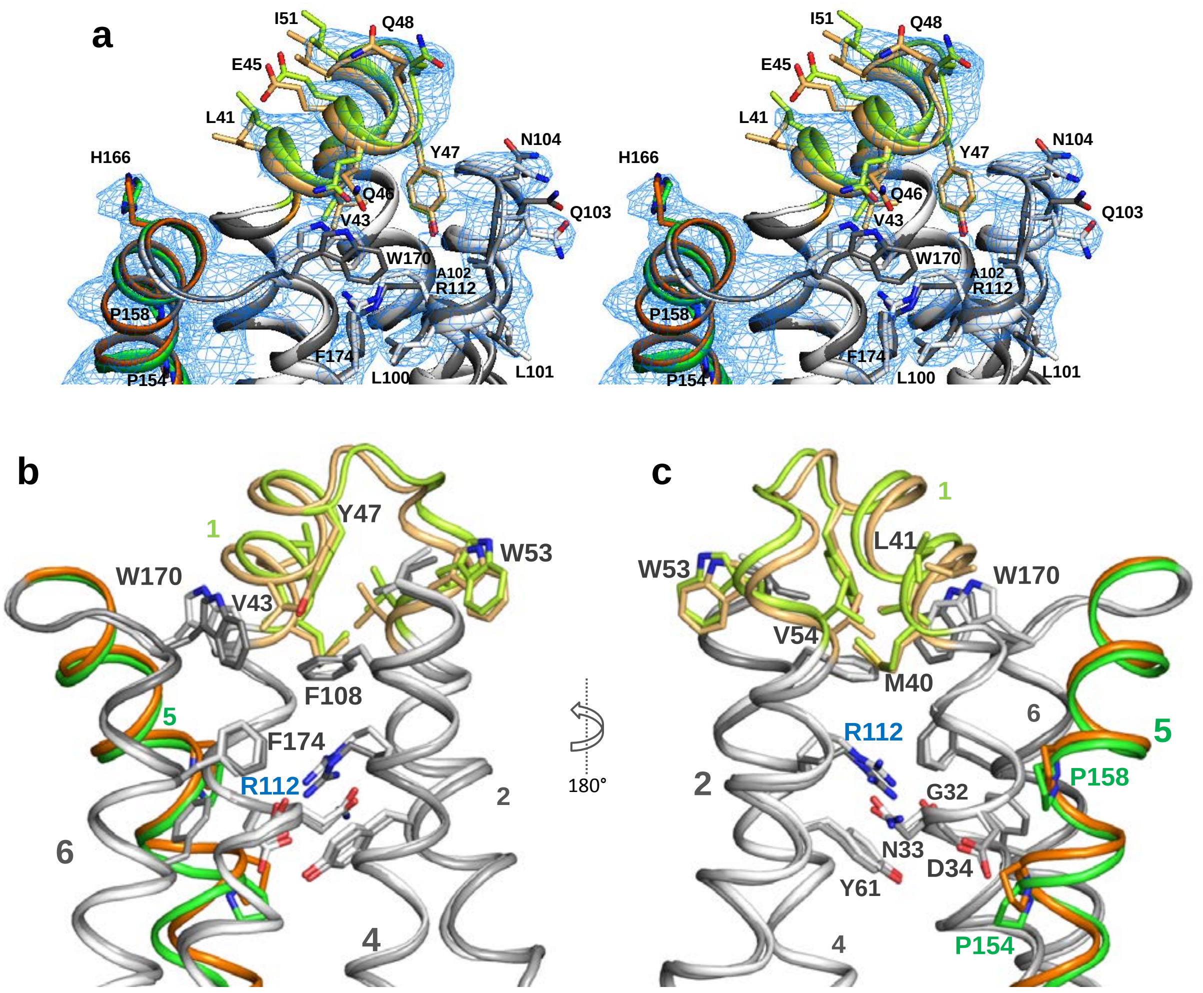

Supplementary Figure 4: Small but significant differences are observed in the hydrophobic core near the periplasmic face of the N-terminal domain. (a) Electron density ($2F_o$-F_c contoured at 1σ, in stereo) for the hydrophobic core in stereo representation. **(b, c)** The core is in contact with the buried guanidinyl moiety of conserved Arg112^{TM4} (motif B), which in turn is connected to Asn33^{TM1}-Asp34^{TM1} by a hydrogen bond network (not shown). View **(b)** from the “left” of **Figure 1** and **(c)** rotated 180° about a vertical axis.

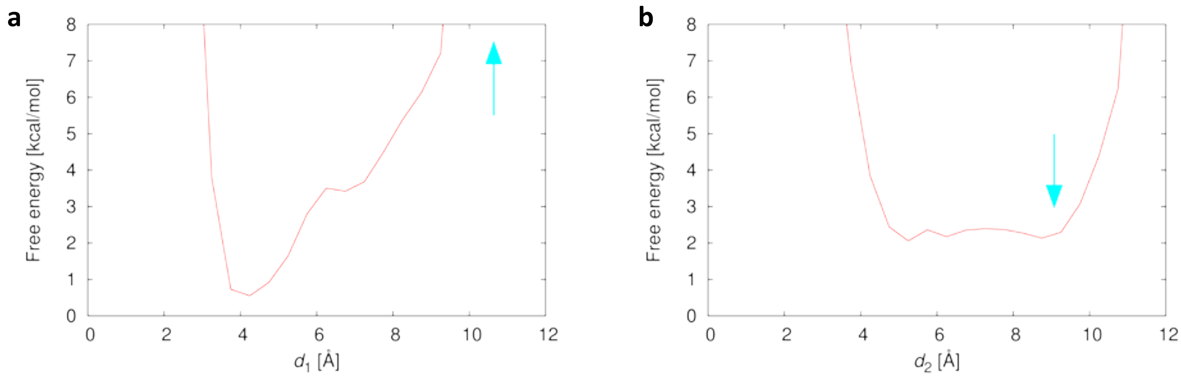

Supplementary Figure 5: Free energy profiles calculated from the distribution of (a) d_1 in the MD run $O_o(E26^-/D34p)$ and (b) d_2 in the MD run $I_f(E26^-/D34p)$. The cyan arrows indicate the d_1 and d_2 values in the initial structures respectively.

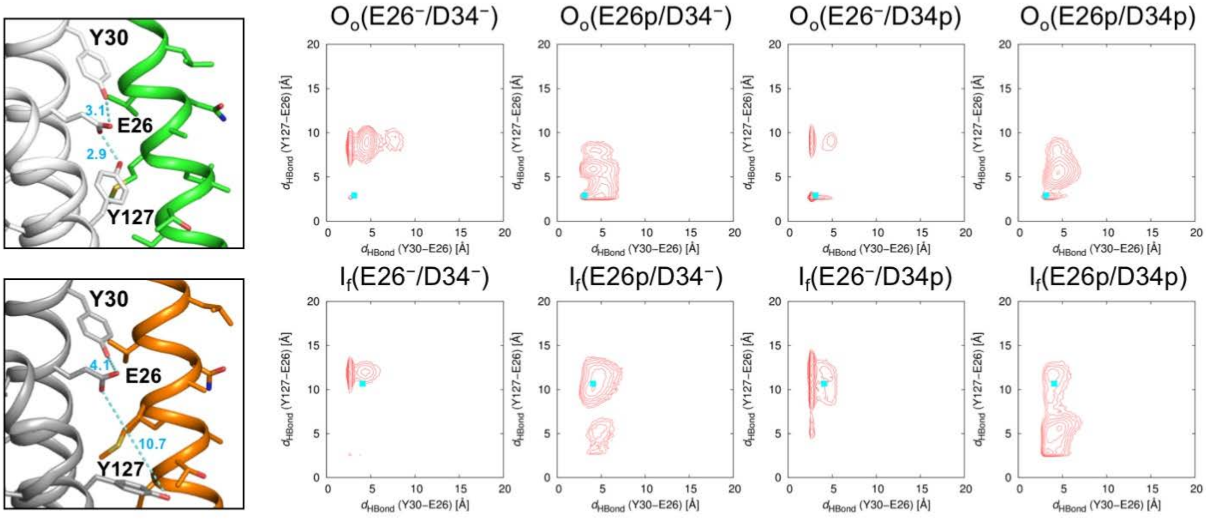

Supplementary Figure 6: Conformational distributions of the transporter during molecular dynamics simulations as a function of starting conformation (O_o vs. I_f) and Glu26/Asp34 protonation states. Plotted are the distances between the Glu26^{TM1} carboxylate and the hydroxyl groups of Tyr127^{TM4} (vertical axes) and Tyr30^{TM1} (horizontal axes). Cyan squares depict distances in the respective initial crystal structures (left).

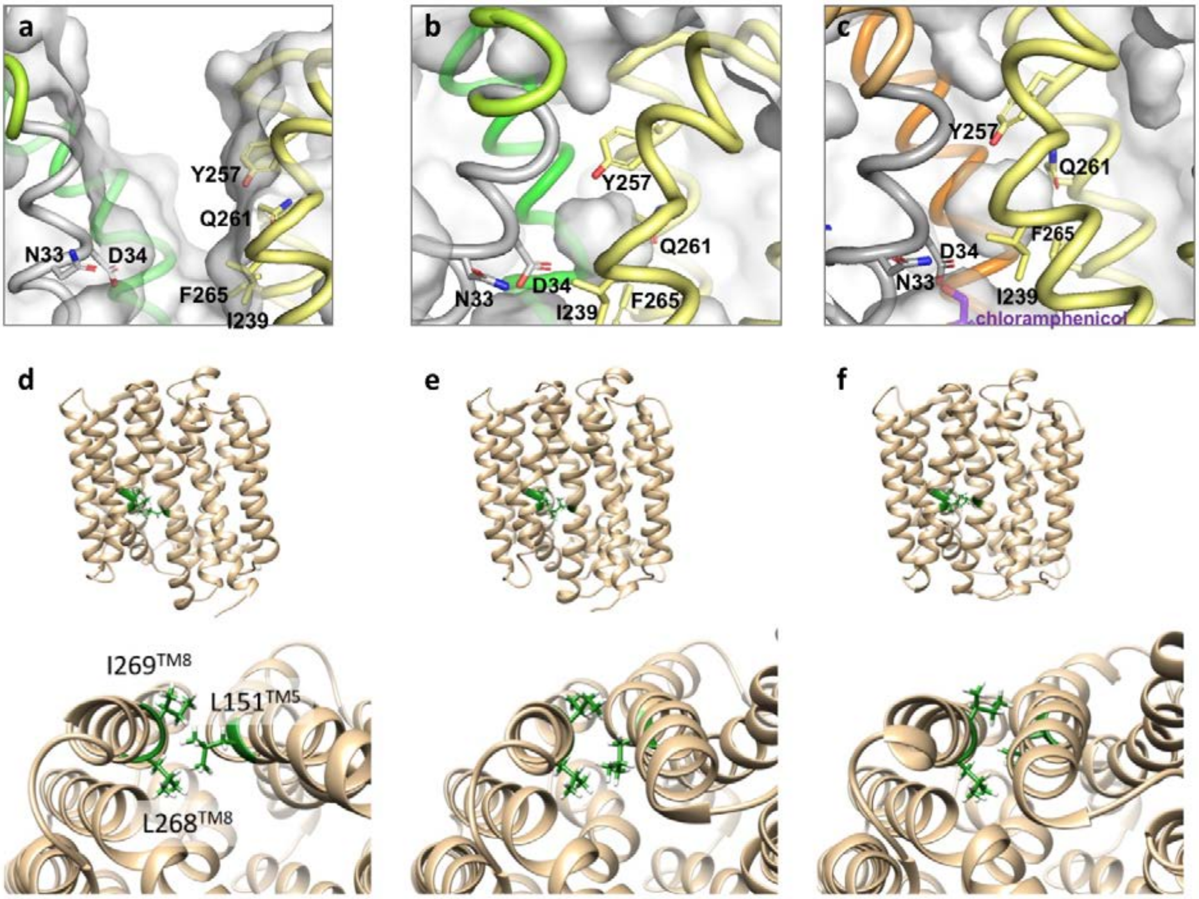

Supplementary Figure 7: Snapshots from the MD simulation trajectories. (a-c) Protonation of Asp34^{TM5} in the O_o state (**a**) results in an occluded state (**b**) in which the Asp34^{TM5} side chain juxtaposes an internal cavity (closed surface) bounded by Tyr257^{TM7}, Gln261^{TM8}, Ile239^{TM7} and Phe265^{TM8}. A similar cavity is found in the chloramphenicol-bound I_f structure (**c**). (**d-f**) TM5 undergoes twisting during the transition from the I_f structure to the occluded state. Lateral and cytoplasmic views of the snapshot structures at 0.5 (**d**), 0.6 (**e**), and 1.0 μs (**f**) of the I_f(E26$^-$/D34^p) runs are shown in the upper and lower panels, respectively. Sidechain atoms of L151^{TM5}, L268^{TM8} and I269^{TM8} are shown in a stick representation. Carbon and hydrogen atoms are colored green and white, respectively.

a

b

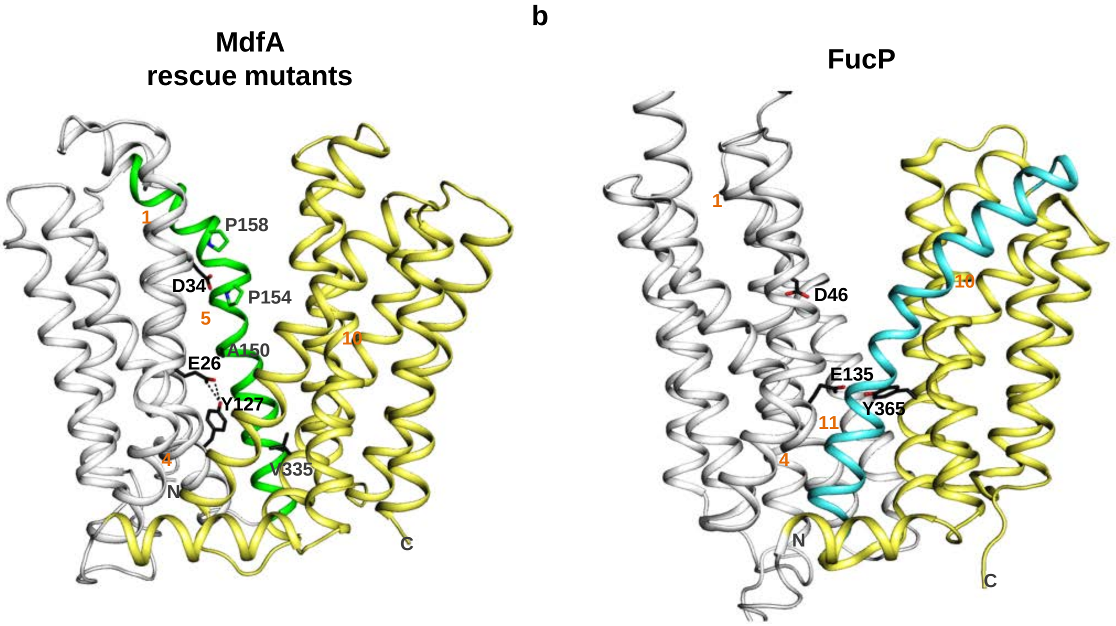

Supplementary Figure 8: Similarities in MdfA rescue mutants and the symporter FucP. (a) Selection for drug transport rescue in cells harboring the otherwise inactive TM1 variants Glu26^{TM1}Thr/Asp34^{TM1}Met and Glu26^{TM1}Thr resulted in the detection of mutants containing the acidic side chains Ala150^{TM5}Glu and Val335^{TM10}Glu[4,5]. These residues would be well positioned to make hydrogen bonds to Tyr127^{TM4} in the outward open structure. **(b)** Recent thermodynamic calculations and molecular dynamic simulations have led in principle to similar conclusions for the L-fucose/H^+ symporter FucP[6]. Using computational methods, it was proposed that protonation of FucP Glu135^{TM4} in TM4 allows surmounting of a ca. 2 kcal mol^{-1} energy barrier between the inward and outward open states. An intermediate state in which TM11 is distorted is postulated, although a causative link between Glu135^{TM4} (de)protonation and TM11 distortion has not been described. Inspection of the FucP structure[7] suggests that Glu135^{TM4} could form a hydrogen bond with Tyr365^{TM10} of C-domain TM10.

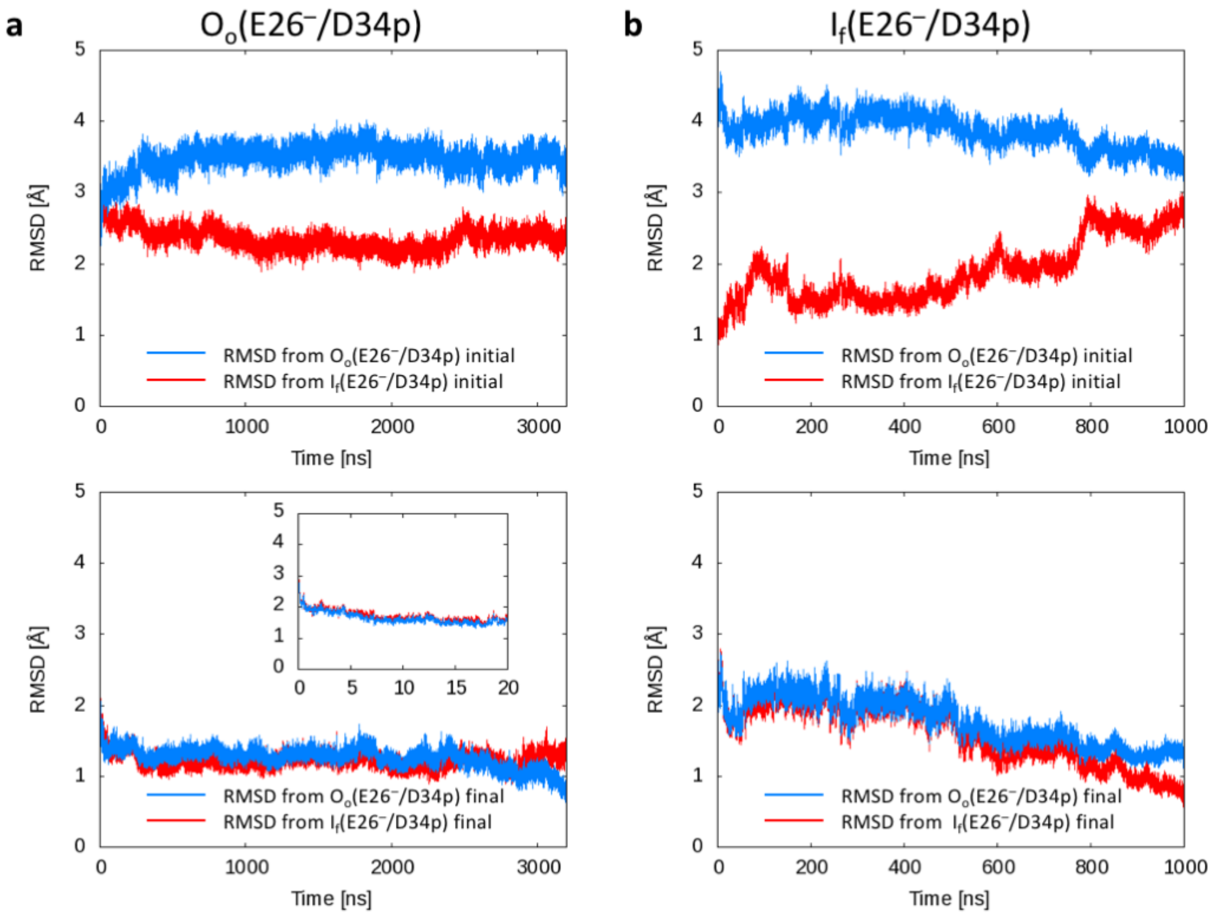

Supplementary Figure 9. Time evolution of the RMSDs from the initial (upper panels) and final (lower panels) structures of the $O_o(E26^-/D34p)$ (blue lines) and $I_f(E26^-/D34p)$ (red lines) MD runs. (**a**) $O_o(E26^-/D34p)$ and (**b**) $I_f(E26^-/D34p)$. The inset shows the RMSDs from the final structures of the MD run $O_o(E26^-/D34p)$ for the time from 0 to 20 ns. RMSDs were calculated for Cα atoms.

Supplementary Table 1. RMSDs of MD simulation snapshots from the crystal structure

Aligned residues[a]	RMSD[b] [Å]
All[c]	2.59 ± 0.27
TM helices[d]	2.46 ± 0.22
N-lobe TM helices[e]	1.45 ± 0.21
C-lobe TM helices[f]	1.08 ± 0.11
Periplasmic halves of TM helices[g]	2.84 ± 0.29
Cytoplasmic halves of TM helices[h]	1.77 ± 0.14

[a]Before calculating RMSD, the coordinates of the Cα atoms of the designated residues of each snapshot structure in the trajectory were aligned with those of the corresponding atoms of the crystal structure.
[b]Mean ± standard deviation.
[c]Residues 14–400.
[d]Residues 14–46, 53–79, 81–101, 105–133, 136–164, 171–191, 219–246, 254–278, 284–308, 313–340, 346–398.
[e]Residues 14–46, 53–79, 81–101, 105–133, 136–164, 171–191.
[f]Residues 219–246, 254–278, 284–308, 313–340, 346–398.
[g]Residues 33–46, 53–63, 99–101, 105–113, 155–164, 171–171, 233–246, 254–264, 298–308, 313–321, 361–387.
[h]Residues 14–32, 64–79, 81–98, 114–133, 136–154, 172–191, 219–232, 265–278, 284–297, 322–340, 346–360, 388–398.

Supplementary Table 2. Primers used for site direct mutagenesis

	Sequence (5' to 3')
E26Q-Fw	CTGTCTGGTGCTTTACCAATTTTCAACCTATATCGG
E26Q-Rv	CCGATATAGGTTGAAAATTGGTAAAGCACCAGACAG
D34N-Fw	TTTCAACCTATATCGGCAACAATATGATTCAACCCGGTATG
D34N-Rv	CATACCGGGTTGAATCATATTGTTGCCGATATAGGTTGAAA
Y127A-Fw	CATTGGCGCTGTGGGAGCGGCCGCAATTCAGG
Y127A-Rv	CCTGAATTGCGGCCGCTCCCACAGCGCCAATG
Y127F-Fw	CATTGGCGCTGTGGGATTTGCCGCAATTCAGG
Y127F-Rv	CCTGAATTGCGGCAAATCCCACAGCGCCAATG
M146A-Fw	TCAAGATCACCGCGCTGGCGGCGAACGTGGCGCTGATTG
M146A-Rv	CAATCAGCGCCACGTTCGCCGCCAGCGCGGTGATCTTGA
W170A-Fw	GTGGATCCATGTGCTGCCCGCGGAGGGGATGTTTGTTTTG
W170A-Rv	CAAAACAAACATCCCCTCCGCGGGCAGCACATGGATCCAC

Supplementary Table 3. Conditions for MD simulations

Run	Initial conformation	State of Glu26^{TM1}	State of Asp34^{TM1}
$O_o(E26^-/D34^-)$	O_o	negatively charged	negatively charged
$O_o(E26^p/D34^-)$	O_o	protonated	negatively charged
$O_o(E26^-/D34^p)$	O_o	negatively charged	protonated
$O_o(E26^p/D34^p)$	O_o	protonated	protonated
$I_f(E26^-/D34^-)$	I_f	negatively charged	negatively charged
$I_f(E26^p/D34^-)$	I_f	protonated	negatively charged
$I_f(E26^-/D34^p)$	I_f	negatively charged	protonated
$I_f(E26^p/D34^p)$	I_f	protonated	protonated

Supplementary References

1. Heng, J. *et al.* Substrate-bound structure of the E. coli multidrug resistance transporter MdfA. *Cell Res.* **25,** 1060–1073 (2015).
2. Liu, M., Heng, J., Gao, Y. & Wang, X. Crystal structures of MdfA complexed with acetylcholine and inhibitor reserpine. *Biophys Rep* **2,** 78–85 (2016).
3. Wilman, H. R., Shi, J. & Deane, C. M. Helix kinks are equally prevalent in soluble and membrane proteins. *Proteins* **82,** 1960–1970 (2014).
4. Sigal, N., Fluman, N., Siemion, S. & Bibi, E. The secondary multidrug/proton antiporter MdfA tolerates displacements of an essential negatively charged side chain. *J. Biol. Chem.* **284,** 6966–6971 (2009).
5. Adler, J. & Bibi, E. Promiscuity in the geometry of electrostatic interactions between the Escherichia coli multidrug resistance transporter MdfA and cationic substrates. *J. Biol. Chem.* **280,** 2721–2729 (2005).
6. Liu, Y., Ke, M. & Gong, H. Protonation of Glu135 Facilitates the Outward-to-Inward Structural Transition of Fucose Transporter. *Biophysical Journal* **109,** 542–551 (2015).
7. Dang, S. *et al.* Structure of a fucose transporter in an outward-open conformation. *Nature* **467,** 734–738 (2010).
