# Supplementary material for: Outward open conformation of a Major Facilitator Superfamily multidrug/H+ antiporter provides insights into switching mechanism
Source: Nat Commun. 2018 Oct 1;9:4005. doi: 10.1038/s41467-018-06306-x (PMC6167325; doi:10.1038/s41467-018-06306-x)
Supplement: Supplementary file 3 — Description of Additional Supplementary Files [file 41467_2018_6306_MOESM3_ESM.pdf]

Description of Additional Supplementary Files

File Name: Supplementary Movie 1

Description: **Conformational changes in TM5 and the hydrophobic core** (c.f. Figures 2, 3 and Supplementary Figures 3 and 4). Fit of O_o and I_f coordinates to $2F_o$-F_c electron density contoured at 1_σ, with morphs between the structures. Movie prepared using PyMOL.
